# Rewriting the valuation and salience of alcohol-related stimuli via memory reconsolidation

**DOI:** 10.1038/tp.2015.132

**Published:** 2015-09-22

**Authors:** R K Das, W Lawn, S K Kamboj

**Affiliations:** 1 Clinical Psychopharmacology Unit, Department of Clinical, Educational and Health Psychology, University College London, London, UK

## Abstract

The transient period of memory instability that can be triggered when memories are retrieved under certain conditions offers an opportunity to modify the maladaptive memories at the heart of substance use disorders (SUDs). However, very well-learned memories (such as those in excessive drinking and alcohol use disorders) are resistant to destabilisation when retrieved or may not destabilise at all. Memory retrieval and intervention procedures that reliably destabilise and update maladaptive motivational memories may help to improve the long-term treatment of SUDs. In 59 hazardous drinkers, we tested a novel retrieval procedure for destabilising well-learned cue-drinking memory networks that maximises prediction error (PE) via guided expectancy violation during retrieval of these memories. This was compared with a retrieval procedure without PE and no-retrieval controls. We subsequently counterconditioned alcohol cues with disgusting tastes and images in all groups and assessed responding to alcohol stimuli 1 week later. Counterconditioning following PE retrieval produced generalised reductions in oculomotor attentional bias, explicit valuation and outcome expectancies in response to alcohol cues 1 week after intervention, evidence of updating of distributed motivational drinking memory networks. These findings demonstrate that well-learned cue-drinking memories can be destabilised and that learning history need not constrain memory destabilisation if PE is maximised at retrieval. Broad rewriting of diverse aspects of maladaptive memory by counterconditioning is achievable following this procedure. The procedure described may provide a platform for the development of novel memory-modifying interventions for SUDs.

## Introduction

In the progression from recreational to hazardous drug use to full-blown addiction, maladaptive motivational memory (MMM) associations are formed that link environmental drug-related stimuli (drug cues), and the availability, intoxicating and rewarding effects of drugs themselves.^1^ These memory traces imbue drug cues with enhanced value, salience and motivational properties by hijacking neural reward circuitry, such that these cues grab attention,^2^ trigger craving and motivate drug seeking-and-using behaviour when encountered.^3, 4^ These lasting mnemonic changes can drive excessive drug use and may be responsible for the high relapse rates in long-term abstainers.^5^ Neutralising MMMs may therefore be instrumental to improving the long-term treatment of substance use disorders (SUDs).

The phenomenon of retrieval-dependent memory destabilisation and restabilisation, known as reconsolidation^6, 7^ entails a novel window of susceptibility of consolidated memories to pharmacological interference^6, 7, 8^ or qualitative updating with new information.^9^ This ‘reconsolidation window' of memory instability has profound implications for treating SUDs as it offers a means of directly weakening or rewriting the MMMs thought to underlie relapse. It may be possible to rewrite the content of destabilised MMMs with more adaptive learning during the reconsolidation window, which could be potentially transformative in the long-term treatment of addiction. This could theoretically overcome the problem of recovery of MMM-mediated drug seeking via renewal (changes in context), reinstatement (re-exposure to drugs) and spontaneous recovery (the simple passage of time) that limit the efficacy of current extinction and exposure-based approaches in SUDs.^10^ There is also some evidence that reconsolidation manipulations may prevent reacquisition of cue-based responding,^11, 12^ which would be highly desirable in preventing relapse.

Extinction training following a single cue-drug memory ‘retrieval' session (which putatively destabilises memory traces), can produce long-term reductions in craving in abstinent heroin addicts.^13^ Despite this promising finding, replication failures are frequent within the paradigm for both fear^14, 15^ and cue-drug learning^16^ and, critically, have not been shown in non-abstinent users exposed to their regular drug-using environments. As memories do not destabilise every time they are retrieved,^17^ null results are likely due either to failure to destabilise memories at retrieval, or to insufficiency of extinction as a corrective post-destabilisation learning modality in MMMs. The effective neutralising of MMMs therefore hinges upon (a) effective destabilisation of MMM networks at retrieval^18^ and (b) appropriate, efficacious forms of post-retrieval corrective learning that reduce motivated drug-seeking processes.

The destabilisation potential of memories is putatively determined by learning history, with aging of memories^19, 20^ and more extensive training^21, 22, 23^ conferring resistance to destabilisation. In human tobacco and alcohol use, MMMs are learned over hundreds of thousands of learning trials in multiple contexts. Such learning history is stronger, by orders of magnitude, than the memory traces shown to reconsolidate in animal and human lab reconsolidation studies (generally tens or hundreds of trials in a single context) and is more extensively varied than the equivalent learning in heroin users. Human alcohol and tobacco MMMs are therefore likely to be highly resistant to destabilisation and it remains to be shown whether such memories undergo reconsolidation at all.

Prediction error (PE), a mismatch between predicted and actual outcomes, has been forwarded as a primary determinant of memory destabilisation at retrieval.^24, 25, 26^ However, in human drug users it is virtually impossible to know what constitutes a PE during MMM retrieval and therefore how to structure reminders to destabilise these memories. Reward learning accrues by minimising PEs over learning episodes,^27, 28^ so PE at retrieval will be low for well-learned memories under normal circumstances. Previous failures to destabilise robust memories may therefore be due to insufficient PE on retrieval. In the lab, where learning is determined by the experimenter, PE can be guaranteed from knowledge of the training history and manipulation of the reminder structure. In naturalistic human drug use, where learning history is unknown, this is not possible. To date, no tests of the sufficiency or necessity of PE for destabilising MMMs within the context of naturalistic human drug use have been conducted and the level of PE generated by reminder procedures in previous studies with drug users was not quantified or manipulated.^13, 29^ Effective procedures for generating PE and destabilising well-learned MMMs at retrieval will be central to assessing the potential of MMM modification in treating SUDs.

Interventions in the reconsolidation window have thus far focused almost exclusively on extinction. However, extinction learning may not be optimal for reducing the valuation and motivational sensitisation to drug cues that is central to the pathology of SUDs, whether conducted post destabilisation or not, as it primarily targets limited and purely associative components of memories,^30^ creating a novel association between a stimulus and lack of reinforcement.^31^ As such, extinction-based therapies are not a particularly effective strategy for reducing drug use *per se*.^10, 32, 33^ Recent experimental research has identified a number of learning-based interventions that have potential for instating adaptive changes in behaviour that may reduce drug use and inform more optimal interventions for use in the reconsolidation window. For instance, training inhibitory responses to alcohol^34^ reduces drinking by attenuating emotional responding to alcohol cues.^35^ Similarly, pairing alcohol-related cues with negative outcomes can reduce motivational and evaluative representations of alcohol.^36^ These remain largely untested within the framework of reconsolidation-update mechanisms, however, and their long-term stability remains untested and they likely require multiple training sessions.^37^ Retraining of MMMs during their reconsolidation would likely benefit from a highly salient and aversive experience during relearning, providing a more emotional and salient experience than extinction,^38^ as engaging emotional responses is a key aspect of successful therapy and may work in concert with reconsolidation to provoke lasting change.^39^ In support of this, counterconditioning procedures have been shown to be more effective than extinction in reducing craving for and consumption of chocolate^33^ and may be more resistant to renewal effects.^40^

These procedures leverage employ disgust, a potent and universally experienced response that is reliably elicited by bitter compounds and certain images,^41^ which leverages a potent evolutionary anti-consumption mechanism, rather than purely reducing the predictability of alcohol from preceding cues. If the aim is to redress aberrantly high motivational influences of Pavlovian alcohol cues relative to alternative rewards, it is intuitively appealing to ‘fight fire with fire', rebalancing the hypervaluation of these cues by pairing them with a highly aversive and de-motivating outcomes by instating cue-evoked disgust^42^ in a counterconditioning procedure.^43^

Here we sought to examine the necessity and sufficiency of PE for destabilising MMMs. We attempted to generate PE without prior knowledge of learning history and in a manner that was clinically practicable among a sample of hazardous beer drinkers. We hypothesised that using explicit instructions to generate the expectancy of beer reinforcement, then unexpectedly withholding beer during retrieval of cue-alcohol MMMs, would create a large PE that would destabilise these memories. Subsequent counterconditioning of beer cues w**i**th disgust-inducing outcomes should then overwrite the reactivated cue-alcohol memories, replacing motivational alcohol associations with disgust responses. This should rebalance the evaluative and motivational status of alcohol cues relative to alternative, non-conditioned rewards, evidenced by (1) reduced valuation and motivational salience of alcohol relative to non-counterconditioned stimuli along with (2) higher cue-induced self-rated disgust propensity following counterconditioned stimuli and (3) lower explicit positive expectancies of alcohol 1 week following intervention.

## Materials and methods

Fifty-nine hazardous, beer-preferring drinkers completed the study. Sample size was chosen to achieve 0.8 power at *α*=0.05, based on a moderate effect size (*r*=0.35) for interaction effects in mixed 3 × 2 analysis of variance (ANOVA). Inclusion criteria were current hazardous drinking defined as a score >8 on the Alcohol Use Disorders Identification Test^44^ but <3 items coded as 3 on the Structured Clinical Interview for DSM-IV; consumption of >3 units for females, >4 units for males on at least 3 days per week; fluent English and normal or corrected-to-normal colour vision. Exclusion criteria were age <18>65, past or current diagnosis of drug or alcohol use disorders, any currently medicated mental health issues, any current major physical health issue; current pregnancy or breastfeeding.

Twenty-four hours prior to Day 1, participants provided written, informed consent and reported their drinking over the past week using the calendar-based timeline follow-back for alcohol,^45^ rated their disgust sensitivity and propensity via the Disgust Propensity and Sensitivity Scale-Revised (DPSS-R)^46^ and their readiness to change their drinking via the Stages of Change Readiness and Treatment Eagerness Scale (SOCRATES)^47^ and expected negative consequences of drinking with the negative alcohol expectancy questionnaire.^48^ Reward and punishment sensitivity were assessed using the behavioural inhibition scales/behavioural activation scale.^49^ Participants were told that they were to take part in an experiment on psychological influences on taste perception and were not informed of the true nature of the experiment until testing was complete. All procedures contributing to this work were approved by University College London ethics committee and complied with the Helsinki Declaration of 1975, as revised in 2008.

### Conditioned stimuli

Four prototypical beer images were selected to act as MMM reactivation cues and subsequently as conditioned stimuli (CS)+s in the counterconditioning task. Multiple, prototypical beer CSs were used to maximise activation of MMM networks and generalisation of the association between beer-related stimuli and disgusting outcomes. The use of single stimuli in reconsolidation paradigms can lead to effects that are highly stimulus specific, rather than generalisable within the category of the reactivated stimulus.^50^

Two novel beer and wine images were used on Day 8 in the liking (picture rating) and attentional bias task to assess generalisation of effects. Two CS−s (a cup of coffee and can of cola) were used in the retrieval and counterconditioning to control for non-associative effects of the procedures. Soft drink images were used as CS−s to rule out generic decrease in liking of consumable stimuli due to anti-consummatory effects of exposure to the disgust UCSs.

Control ‘no-reactivation' cues presented during the retrieval stage in the Control group depicted four orange juice-related images. The beer and orange juice cues were equated as much as possible to minimise any effects that were not specific to the reactivation manipulation.

### Retrieval (Day 1)

**Figure 1:**
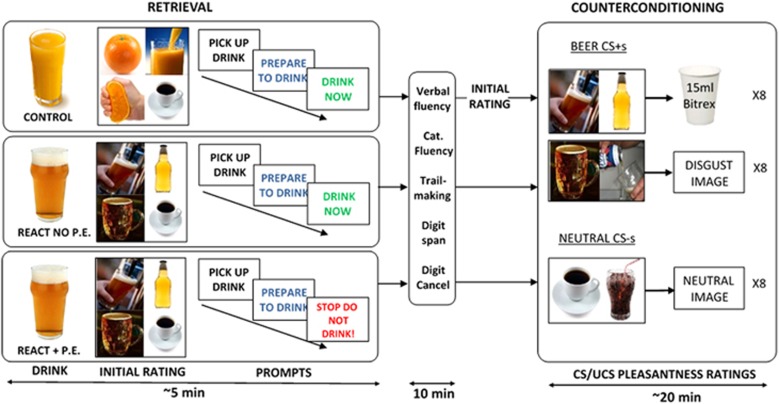
Schematic diagram of retrieval/counterconditioning procedure. Counterconditioning is identical for all groups. All groups initially are given a drink and told they will consume it after rating some pictures. In the REACT no PE and REACT+PE groups this is 150 ml beer, in the Control group 150 ml orange juice. Control and REACT no PE consume the drink as expected. In REACT+PE, it is withheld at the last moment. Note the Control group rate the beer images at the beginning of counterconditioning (with no time delay). CS, conditioned stimuli; PE, prediction error; REACT, reactivation.

The procedure in the three experimental groups differed only in the nature of the MMM ‘reactivation (REACT) session' 10 min prior to counterconditioning on Day 1. Participants were randomly assigned to group based on their time of entry into the study using a random number generator. The Control group (*n*=20) received a 150 ml glass of orange juice and were told they would consume this, according to on-screen prompts, after rating pictures of orange juice for pleasantness. The prompts consisted of three consecutively displayed screens saying ‘PICK UP DRINK', ‘PREPARE TO DRINK' and ‘DRINK NOW'. They rated the four orange juice-related pictures, two control soft drink images (cola and coffee) and then consumed the juice in time with the on-screen prompts (see Figure 1). The REACT no PE (*n*=20) and REACT+PE (*n*=20) groups were given a 150 ml glass of (alcohol-free, unknown to participants, to avoid alcohol effects on subsequent task performance) beer and instructed they would drink it all, according to on-screen prompts, after alcohol pictures were rated for pleasantness (an index of beer cue valuation). They then rated the four prototypical beer cues and the two control soft drink cues.

The REACT no PE group then consumed the beer according to the prompts, as expected, recapitulating the reinforcement in a typical drinking episode. In REACT+PE group, instead of reading ‘DRINK NOW' the final on-screen prompt unexpectedly instructed the participants: ‘STOP, DO NOT DRINK' and to put down the glass just prior to expected consumption (Figure 1). This procedure was designed to maximise expectation of alcohol reward and therefore negative PE when this was withheld. Verbal fluency and category fluency, digit span,^51^ trail-making^52^ and digit cancellation tasks were then performed immediately to fill 10 min following the reactivation procedures. These high working-memory load tasks were chosen to ensure disengagement of cue-beer memory networks prior to counterconditioning.^53^ The 10-min space between retrieval and counterconditioning was based on previous studies successfully employing this space in prototypical retrieval-extinction paradigms.^9, 12, 13^

### Counterconditioning

The Control group rated the beer cues at the start of counterconditioning, after the distractor tasks, to provide a baseline pleasantness rating for these stimuli in this group so that number of exposures to all stimuli were identical across groups.^16^ Counterconditioning paired the four beer cues (CS+s) presented during the reactivation session (or at the start of the task in the Control group), with either disgusting pictures from the International Affective Picture System^54^ or 15 ml 0.067% Bitrex solution (McFarlan Smith, Edinburgh, UK) UCSs. The eight drink UCSs were prepared in opaque plastic cups that participants had to pick up and consume in their entirety when the words ‘drink now' appeared on screen. Each empty cup was replaced with a full one and the number of remaining cups was hidden from the participant. The two control soft drink cues (CS−s) were paired with neutral International Affective Picture System (IAPS) pictures (Figure 1). Counterconditioning was designed in an attempt to maximise the differentiation of outcome valence across stimulus categories and engender general rule learning of the form ‘soft drink stimulus→neutral outcome' and ‘beer stimulus→disgusting/aversive outcome', with the aim of generalising learned aversion to alcohol stimuli. As such, a 100% reinforcement schedule was employed. Soft drink CSs with were never paired with disgusting outcomes, as doing so would bias learning towards individual CS-specific learning.^50^ Each of the CSs were presented four times during the task in a standard pseudo-randomized order such that eight Bitrex and eight IAPS UCSs were delivered over the entire task. Pleasantness ratings of CS+s, CS−s and UCSs were collected online throughout counterconditioning.

### Test (Day 8)

On Day 8, participants re-rated all CSs along with novel beer and wine cues. Attentional allocation, an index of motivational salience, to these images was assessed by tracking eye movements in a visual probe task where all CSs were paired with matched control images. Participants then completed the Alcohol Craving Questionnaire (ACQ-NOW),^55^ DPSS-R and timeline follow-back to assess cue-induced craving, disgust and drinking since Day 1. Finally, participants were asked to write down what they believed to be the purpose of the study to assess any demand effects resulting from the belief that the study had an interventional nature. Following the completion of testing, participants were fully debriefed as to the true purpose of the study.

### Statistical approach

Data analysis was performed using IBM SPSS version 21 for Windows (IBM, Armonk, NY, USA). All data were checked for normality, homogeneity of variance and sphericity (for repeated-measures with *k*>2 comparisons). Where homogeneity of variance was violated in one-way ANOVA, Welch's F test is reported. Where sphericity was violated, the Huynh–Feldt correction was applied to the degrees of freedom and significance levels. Uncorrected degrees of freedom are reported here, with corrected *P*-values. Any outliers >3 s.d. away from the sample mean for that variable were Winsorised to a score 3 s.d. from the mean. Overall, <3% data were Winsorised in this way. Analyses were run with and without this procedure and this did not affect the pattern of the results in any substantial way. For single time-point measurements, one-way ANOVA was used to assess group differences, and for repeated measurements mixed ANOVA with a between-subjects factor of Group was used. Significant *k*>2 main effects and interactions in omnibus ANOVAs were investigated with independent or paired-samples *t*-tests on marginal means, where appropriate. For attentional bias data, significant interactions were assessed by examining effects across Groups, as the appropriate within-subjects comparison between alcohol and neutral stimuli is incorporated into the attentional bias score. For liking data, interactions were assessed by comparing stimulus types within subjects, as we feel the relative balance of drug and non-drug reward valuation within individual is more informative than the absolute level of scores.^56^ Significance values for *post hoc* tests are Bonferroni-corrected to control Type I error (Supplementary Materials).

## Results

Descriptive statistics of baseline demographic and drinking measures are given in Table 1, along with statistical tests of group differences. Groups did not differ on any of these measures.

### Retrieval/counterconditioning

Counterconditioning of stimuli was assessed via a 2 (CS Type: beer picture CS+s, neutral picture CS−s) × 5 (Trial: Baseline, Trial 1–4) × 3 (Group: Control, REACT+PE, REACT no PE) mixed ANOVA on pleasantness ratings. CS pleasantness ratings during the retrieval phase were the baseline ratings in the REACT+PE and REACT no PE groups and ratings at the beginning of the counterconditioning were the baseline in the Control group. Main effects of CS Type (F(1,56)=7.842, *P*=0.007, *η*_p_^2^=0.123) and Trial (F(4, 168)=3.026, *P*=0.041, *η*_p_^2^=0.051) and critically a CS Type × Trial interaction (F(4, 224)=9.902, *P*<0.001, *η*_p_^2^=0.15) were found. Bonferroni corrected, planned follow-up pairwise comparisons of the interaction found no significant difference between liking of beer and soft drink beverage CS−s (cola and coffee) at baseline (*t*(58)=0.29, *P*>0.5), but greater liking of CS−s from Trial 2 of conditioning (*t*(58)=3.38, *P*=0.001, *r*=0.41), subsequently (Trial 3 *t*(58)=3.93, *P*<0.001, *r*=0.46; Trial 4 *t*(58)=3.52, *P*=0.001, *r*=0.42) There were no Group differences in conditioning of CSs during counterconditioning (Group × CS × Trial interaction F(8, 224)=1.28, *P*=0.256, *η*_p_^2^=0.004).

### Day 8 CS valuation

**Figure 2:**
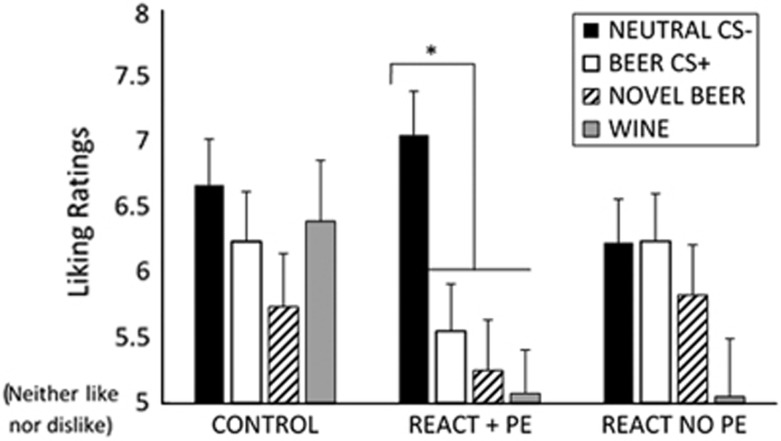
Reduced liking of alcohol stimuli produced by counterconditioning following MMM reactivation with prediction error. Bars represent mean±s.e.m. A score of five denotes neither liking nor disliking of the stimuli. CS, conditioned stimuli; MMM, maladaptive motivational memory; PE, prediction error.

A 3 (Group) × 4 (Picture Type; Beer CS+s, Neutral Cs−s, Novel beer, Wine) mixed ANOVA assessed ratings of CSs from Day 1, along with ratings of novel beer pictures and novel wine pictures. Novel stimuli were included to assess generalisation of liking effects to unconditioned alcohol stimuli. A Picture Type main effect (F(3, 168)=8.44, *P*<0.001, *η*_p_^2^=0.131) and Group × Picture Type interaction was observed (F(6, 168)=2.622, *P*=0.028, *η*_p_^2^=0.086), with no main effect of Group (F(2, 56)=1.027, *P*=0.365, *η*_p_^2^=0.035). To assess the interaction, the simple effect of Picture Type within each Group was examined. This found a highly significant effect of Picture Type in REACT+PE (F(3,57)=8.841, *P*<0.001, *η*_p_^2^=0.318), with non-significant effects of Picture Type in the Control (F(3,54)=2.336, *P*=0.109, *η*_p_^2^=0.115) and REACT no PE Groups (F(3,57)=2.653, *P*=0.086, *η*_p_^2^=0.123). Planned, Bonferroni-corrected comparisons of alcohol stimuli to neutral CS ratings showed relatively reduced valuation of all alcohol stimuli in the REACT+PE group, with lower liking for previously counterconditioned beer CS+s (*t*(19)=3.27, *P*=0.011, *r*=0.6), as well as new beer (*t*(19)=3.91, *P*=0.001, *r*=0.67) and wine stimuli (*t*(19)=3.72, *P*=0.003, *r*=0.65) relative to neutral stimuli. No differentiation in liking between neutral and alcohol stimuli was observed in the other groups. These data are shown in Figure 2. As a secondary analysis, the main effect of Group was assessed for each Picture Type. This demonstrated that the groups did not differ overall in their ratings of the different pictures (all F (2, 58)<1.6, *P*s>0.2) except for wine pictures (F(2, 58)=3.16, *P*=0.05, *η*^2^=0.1), where a marginal difference was seen, but *post hoc* tests did not survive Bonferroni correction (*P*>0.1).

### Attentional bias to CSs

**Figure 3:**
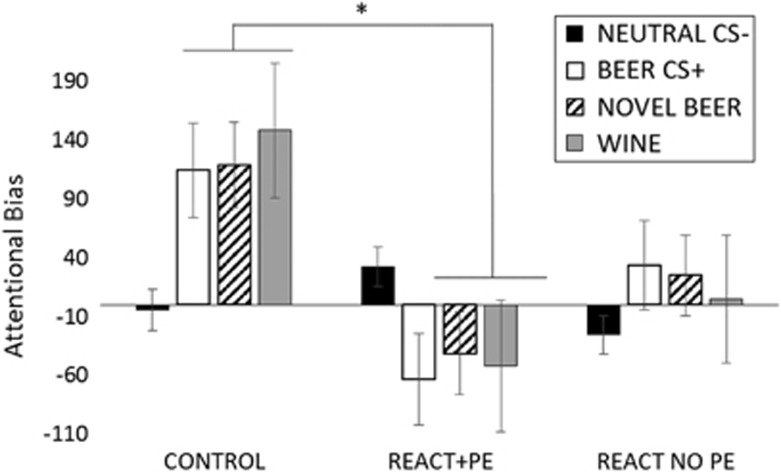
Abolition of attentional bias at test in REACT+PE group. Attentional bias=dwell time on target image (CSs and novel alcohol cues)—dwell time on matched neutral image. Bars represent mean ±s.e.m. CS, conditioned stimuli; PE, prediction error; REACT, reactivation. Asterisk significant at *P*<0.05.

Two participants' eye tracking data were discarded (one from the control group and one from the REACT+PE group) due to insufficient fixations on any images during the task. A 3 (Group) × 4 (Picture Type; Beer CS+s, novel beer, wine, neutral) mixed ANOVA was performed on attentional bias scores in the visual probe task. These were calculated as target image dwell time minus matched control image dwell time. A Group main effect (F(2, 54)=4.768, *P*=0.012, *η*_p_^2^=0.15) was found, driven by lower overall attentional bias in REACT+PE than the Control group (*t*(35)=3.03, *P*=0.011, *r*=0.46), with no difference between REACT+PE and REACT no PE (*t*(37)=1.01, *P*=0.96, *r*=0.17) or REACT no PE and Control (*t*(35)=2.08, *P*=0.128, *r*=0.33). The Group effect was subsumed under a Group × Picture Type interaction (F(6, 162)=3.293, *P*=0.013, *η*_p_^2^=0.109), indicating an oculomotor aversion to alcohol (Beer CS+s *t*(35)=3.19, *P*=0.007, *r*=0.47; NEW BEER *t*(35)=3.16, *P*=0.008, *r*=0.47 WINE *t*(35)=2.5, *P*=0.046, *r*=0.39) but not soft drink CS pictures (*t*(35)=1.5, *P*=0.422, *r*=0.25) in the REACT+PE group, relative to the Control group (see Figure 3). There was no overall effect of Picture Type (F(3, 162)=0.796, *P*=0.45, *η*_p_^2^=0.015).

### Cue-induced disgust and craving

A 2 (Day: Baseline, Day 8) × 2 (Subscale: sensitivity, propensity) × 3 (Group) ANOVA performed on DPSS-R rated disgust found main effects of subscale (F(1,56)=78.14, *P*<0.001, *η*_p_^2^=0.583) and a Day × Subscale × Group interaction (F(2,54)=5.189, *P*=0.009, *η*_p_^2^=0.161). Analyses by Group found the interaction between Day and Subscale was significant only in the React+PE group, with this group showing an increase in the disgust propensity subscale from baseline to post-picture rating on Day 8 (*t* (19)=2.81, *P*=0.006, *r*=0.54, Bonferroni corrected), indicating stronger recall of the aversive reinforcement used during the counterconditioning task.

As the retrieval counterconditioning was designed to update outcome expectation in response to beer cues, we conducted planned analysis on the expectancy subscale of the ACQ-NOW on Day 8 (note that the ACQ was not completed on Day 1 to avoid interference with the memory reactivation procedure). This showed an effect of GROUP (F(2, 57)=3.66, *P*=0.032, *η*^2^=0.13) driven by more negative expectancy of alcohol-related outcomes in the REACT+PE group than the Control Group (*t*(36)=2.81, *P*=0.008, *r*=0.42, Bonferroni corrected). Group differences on the other subscales were not observed.

### Changes in drinking

All groups reduced their self-reported drinking from baseline to Day 8 (F(1, 56)=4.99, *P*=0.03, *η*_p_^2^=0.082), indicating that counterconditioning of itself may be a useful intervention in reducing alcohol consumption. No main effects of Group (F(2, 53)=1.924, *P*=0.156, *η*_p_^2^=0.068) or Group × Time interaction (F(2, 53)=0.098, *P*=0.907, *η*_p_^2^=0.004) were observed, however.

### Exploratory analysis

The observed overall reduction in drinking could be due to non-specific effects such as the Hawthorne effect, regression to the mean or increased drinking awareness. To explore the variance in beer drinking attributable to changes in beer CS liking (that is, the intervention), baseline and Day 8 (test) ratings of beer CSs were correlated with post-intervention alcohol consumption. These correlations are given in Table 2. In the REACT+PE only, Day 8 CS liking ratings predicted a significant amount of variance in post-intervention drinking (*r* (20)=0.589, *P*=0.006). These correlations were not significant in the Control or REACT no PE groups (ps>0.14). Further, in the Control drinking (*r* (19)=0.528*, P*=0.02) and REACT no PE (*r* (20)=0.758*, P*<0.01) groups, there was a significant correlation between baseline and Day 8 CS ratings, indicating return to pre-intervention valuation, in line with ANOVA data. Such a correlation was not seen in the REACT+PE group.

### UCSs

Ratings of unconditioned disgust to the UCSs (IAPS disgust pictures, Bitrex or neutral pictures) were assessed with 3 (UCS) × 3 (Group) × 4 (Trial) mixed ANOVA. A large effect of UCS (F(2,112)=156.65, *P*⩽0.001, *η*_p_^2^=0.737) was observed, indicating unconditioned aversion to the Bitrex (*t*(58)=13.6, *P*<0.001, *r*=0.87) and picture UCSs *t*(58)=15.79, *P*<0.001, *r*=0.9) relative to the neutral pictures.

### Awareness of study purpose

None of participants correctly guessed the true purpose of the study according to the free-report measures. The most common interpretation of the study was that we were testing whether images of pleasant drinks could make unpleasant tastes more pleasant.

## Discussion

The apparent resistance of old and strongly trained memories to destabilisation poses a serious challenge to the use of reconsolidation to ‘fix' maladaptive memories in SUDs. Here we observed reliable and comprehensive MMM restructuring in hazardous drinkers after a memory reactivation procedure including a PE, consistent with reconsolidation-update effects. This shows that very robust alcohol MMMs can reconsolidate and outline a preliminary means for producing MMM destabilisation. These findings are highly encouraging, as alcohol MMMs represent some of the most robust and destabilisation-resistant maladaptive memory traces in psychiatric disorders.^57, 58^ The findings also provide some support for the importance of PE in destabilising clinically relevant MMMs. We attempted to engender PE through explicitly-guided expectancy violation, instead of inferring it from learning history. This violation sufficiently destabilised MMM networks such that subsequent counterconditioning apparently rewrote the valuation and motivational salience of alcohol cues relative to neutral cues, with an associated reduction in positive expectancies of alcohol. As the motivational status of drug and non-drug rewards are thought to be central in the pathogenesis of SUDs and relapse in addicts^56^ and typically resistant to intervention, their observed reduction via MMM updating is extremely promising for the use of this approach in the treatment of SUDs.

Importantly, the reactivation-dependent reductions in cue valuation and salience generalised to novel alcohol cues. Reconsolidation-interference effects to date have typically been specific to discrete reactivated memory traces, with second-order associations unaffected.^59^ Such specificity in updating effects would potentially limit the efficacy of rewriting individual MMMs in reducing drug seeking-and-using. However, we demonstrate that generalised updating of MMM networks can be achieved by destabilising and counterconditioning only a handful of prototypical drinking cues. The level of generalisation observed is likely a function of the corrective learning employed. The current study employed a counterconditioning paradigm that attempted to maximise within-class stimulus generalisation by only pairing beer stimuli with disgusting outcomes. As such, the simplest and most parsimonious rule to learn to predict cue outcome was ‘alcohol→disgusting outcome' and ‘soft drink→neutral outcome'. If memory destabilisation potentiated the retention of this rule learning by writing it over existing memory networks, this would explain the generalisation of effects to novel cues in the REACT+PE group at test.

This extends preclinical research showing that responding to multiple outcome-predictive cues can be reduced by blocking reconsolidation following reactivation of one of these cues, if the cues are interconnected.^60^ That is, activation of diffuse memory networks by pattern completion from subsets of inputs can engender widespread destabilisation of pathological memory traces. The high degree of inter-connectedness, hippocampal independence and trace dominance of MMMs in humans may therefore work in favour of reconsolidation-based interventions by allowing generalisation of intervention effects among MMM networks. The degree of generalisation during memory updating likely depends on the number of reactivated and counterconditioned cues, their similarity to each other and their perceptual similarity to actual cues conditioned during drug use history. Intervention modalities such as bias modification or response inhibition training may also vary in their degree of generalisability. Such parameters will need to be investigated and optimised in future research to assess the levels of generalisation that are achievable.

Evidence for intermediate-level reduced motivational salience of alcohol cues was observed in the REACT no PE group, with no significant differences between this and the PEACT+PE group. This may be interpreted as evidence that PE is not as critical as believed in destabilising MMMs. However, this reduction was not significantly different from the Control group. Further, it is possible that some level of PE was experienced in this group, due to the novelty of drinking in an experimental context, the relatively small amount of beer available or expected versus perceived pleasantness of the beer used. This is in line with the previous literature that has shown variable amnesia following putatively variable PE^61^ and is in line with the proposal that the level of drug memory destabilisation that occurs at retrieval is proportional to the size of PE experienced. However this is not something that has been observed in the fear conditioning literature, where PE can be more easily quantified.^25, 62, 63^

Attempting to maximise PE may still be the optimal approach to MMM destabilisation, as the strongest effects were consistently seen for the REACT+PE group in the current study. Alternatively, there may be different requirements for PE for different levels of reward memory.^64^ Changes in explicit valuation may require quantitatively or qualitatively different PE at retrieval than lower-level oculomotor effects, although the reason why this might be the case is currently unclear. A fruitful avenue of future research will therefore be intro procedures that produce different levels and forms of PE at retrieval.

An alternative explanation for the current findings is that generating PE-enhanced encoding of counterconditioning through induction of stress or arousal. The level of arousal and/or stress induced by the omission PE in the current study was not quantified so it cannot easily be disentangled from an effect of PE on memory destabilisation. Previous research has found that glucocorticoid-mediated stress enhances consolidation, but impairs reconsolidation.^65^ As the current intervention relied on the successful reconsolidation of MMMs in their updated form, increased stress from reward omission may have resulted in smaller intervention effects although non-linear relationships are the norm in this field further complicating this interpretation. If new learning/consolidation mechanisms were targeted, increased arousal or stress from reward omission could have potentiated such learning, leading to the results observed here. Given the very short-term effects of even drug-enhanced corrective learning interventions for MMMs,^32, 66^ we feel the striking results observed here are unlikely to be explained by enhanced consolidation of new learning. It is possible that arousal directly modulates the destabilisation potential of memories, although no research exists, to our knowledge, examining this possibility in the context of drug use. Although we believe that reconsolidation update is the most likely explanatory mechanism for the results observed here, further research will be required to rule out these alternative mechanisms. Collecting arousal ratings at retrieval and developing reliable independent metrics of PE and memory destabilisation at retrieval will go a long way towards resolving this issue.

The use of post-destabilisation disgust images and Bitrex counterconditioning is relatively novel, and consistent with previous research utilising counterconditioning^33, 40^ we found evoking core disgust to be an effective way of targeting motivational and evaluative components of alcohol MMMs. In the laboratory context (an atypical environment in which to drink), extinction is unlikely to be a highly salient or memorable reinforcer if it does not engage affective mechanisms. Previous human research has shown efficacy of reconsolidation-based interventions only in abstinent inpatient drug users, a sample for whom drug availability and motivation to abstain are very different to the current sample and non-abstinent individuals with SUDs. During a retrieval-extinction intervention in this group^13^ drug cues in the absence of drug produced powerful craving and therefore created a highly salient learning experience. However, pure reward omission may not sufficiently affect motivational and affective components of alcohol and other MMMs to produce therapeutic change,^30^ particularly in a hazardous, but not addicted sample.

For these reasons, we believe disgust-based counterconditioning warrants further attention as a post-destabilisation learning intervention. The current findings may also be particularly pertinent for improving the efficacy of historical and current aversion therapies (for example, disulfiram), which may be greatly potentiated via combination with memory destabilisation. Indeed, engagement of reconsolidation mechanisms explain some of the observed variance in the long-term efficacy of behavioural interventions.^39^ One limitation of gustatory disgust-based corrective learning is that it may only be effective for drugs that are ingested orally, since evoked ‘core' disgust tends to operate most effectively as an oral anti-consummatory or potentially emetic mechanism.^67^ Other experimental interventions showing short-term efficacy, such as inhibitory training^34^ or cognitive bias modification,^68^ therefore also warrant investigation within the context of reconsolidation update, as this could potentially increase the longevity of their beneficial effects and extend the utility of the approach to many drugs of abuse. Tailoring corrective learning to the modality of drug consumption will be a key in developing interventions that leverage reconsolidation to update MMMs.

Importantly, our participants expressed no desire to reduce their drinking, and although all groups reduced their drinking over the course of the study change in drinking was related to cue valuation and salience only in the REACT+PE group. This suggests that non-specific effects, such as the Hawthorne effect and regression to the mean, must have driven drinking reductions in the control and REACT no PE groups to a larger extent than the REACT+PE group, given the non-differential group effects on absolute drinking reduction. Naturalistic experience of conditioned disgust responses when drinking in the time between Day 1 and Day 8 may have contributed to reduced motivational status of alcohol stimuli on Day 8. If memory destabilisation prior to counterconditioning overwrote existing memory traces, it would be expected that disgust conditioned responses would be retrieved when beer cues were encountered naturalistically, causing further experience-dependent changes in appraisal of those stimuli. This would explain both the correlation between reduced drinking and reduced cue valuation in the REACT+PE group and the increased self-reported disgust propensity in this group. If this were the case, longer-term reductions in drinking would be expected in the REACT+PE group. Although we attempted to collect longer-term follow-up data, attrition was too high to be able to conduct any statistical analysis.

Taken together, these results show that MMM destabilisation is possible without knowledge of learning history through manipulation of the nature of memory retrieval, and that counterconditioning following this shows promise as an intervention for reducing drinking in hazardous drinkers. MMM destabilisation/counterconditioning therefore shows promise as an intervention in hazardous and binge-drinking populations, with the potential to prevent the transition to more severe levels of AUD in those who do not express concern about their drinking.

## Figures and Tables

**Table 1:** Drinking and intervention-relevant demographic self-report measures with statistical tests of differences for baseline data. Data represent mean ±s.d

	*Control (*N*=19)*	*REACT+PE (*N*=20)*	*REACT no PE (*N*=20)*	*F(2, 56)*	P*-value*
*Baseline measures*					
* *AUDIT	14.58±4.72	15.8±4.05	15.1±4.75	3.6	0.7
* NAEQ*					
* *Same day	40.74±7.87	41.75±9.73	42.35±7.59	0.18	0.84
* *Next day	36.21±9.37	36.1±11.43	36.65±8.94	0.2	0.98
* *Continued	29.47±13.04	29±8.52	31.1±6.42	0.26	0.77
* SOCRATES*					
* *Recognition	11.32±3.16	11.2±3.81	13.2±3.38	2.09	0.13
* *Ambivalence	8.16±3.56	8.25±3.02	10.3±3.21	2.72	0.08
* *Taking steps	17.63±7.15	14.9±5.7	17.25±5.9	1.1	0.34
* DPSS-R*					
* *Sensitivity	12.68±3.61	14.8±6.69	12.6±3.03	1.37	0.26
* *Propensity	18.16±2.19	16.9±3.32	16.7±2.64	1.58	0.22
* *Total	30.84±4.86	29.9±5.44	29.3±4.49	0.48	0.62
* *AGE	23.16±7.49	21.5±1.73	23.15±7.44	0.48	0.62
* BIS/BAS*					
* *Drive	11.68±1.95	11.95±1.54	10.8±1.82	2.3	0.11
* *Fun seeking	13.68±2.26	13.25±1.25	13.1±1.97	0.51	0.6
* *Reward	17.63±1.67	17.45±1.93	16.95±1.54	0.83	0.44
* *BIS	20.58±3.01	21.15±3.31	19.75±3.67	0.89	0.42
* DRINKING previous week*					
* *Daily pints beer	1.36±1.15	1.31±0.75	1.86±1.27	1.52	0.23
* *Daily spirits 25ml	0.74±.0.7	1.26±0.86	1.68±1.79	2.37	0.1
* *Daily wine 175ml	0.5±0.67	0.65±0.71	0.23±0.34	2.86	0.07

*Day 8 measures*					
* DRINKING previous week*					
* *Daily pints beer	1.05±0.95	1.09±0.93	1.64±1.21		
* *Daily spirits 25ml	0.68±0.85	0.94±0.98	1.53±2.16		
* *Daily wine 175ml	0.5±0.58	0.52±0.64	0.51±0.66		
* ACQ Day 8*					
* *EMOT	3.65±1.55	3.5±1.39	3.33±1.37		
* *PURP	4.65±1.11	4.28±0.67	4.55±1.22		
* *COMP	2.77±1.83	2.08±0.94	2.6±1.34		
* *XPECT	4.75±0.96	3.87±1.01	4.08±1.2		
* DPSS-R Day 8*					
* *Sensitivity	12.46±4.61	12.9±4.59	13.77±3.65		
* *Propensity	18.4±3.56	18.85±4.17	17.01±3.42		
* *Total	30.85±7.71	31.75±7.62	30.78±5.45		

Abbreviations: ACQ, alcohol craving questionnaire; AUDIT, alcohol use disorders identification test; BIS/BAS, behavioural inhibition scales/behavioural activation scale; DPSS-R, disgust propensity and sensitivity scale- revised; NAEQ, negative alcohol expectancy questionnaire; PE, prediction error; REACT, reactivation; SOCRATES, stages of change readiness and treatment eagerness scale.

**Table 2:** Correlations of CS liking ratings and post-intervention drinking across experimental groups

	*Control*		*REACT+PE*		*REACT no PE*	
	*Baseline CS rating*	*Day 8 CS rating*	*Baseline CS rating*	*Day 8 CS rating*	*Baseline CS rating*	*Day 8 CS rating*
Day 8 CS rating	0.528^a^	—	−0.03	—	0.758^b^	—
Post-intervention daily drinking	−0.008	−0.109	0.274	0.589^b^	0.337	0.286

a Significant at *P*<0.05.

b Significant at *P*<0.01.

CS, conditioned stimuli; PE, prediction error.
