# Supplementary material for: Rewriting the valuation and salience of alcohol-related stimuli via memory reconsolidation
Source: Transl Psychiatry. 2015 Sep 22;5(9):e645–. doi: 10.1038/tp.2015.132 (PMC5068809; doi:10.1038/tp.2015.132)
Supplement: Supplementary Information [file tp2015132x1.docx]

**Materials and Methods:**

**Participants:**

Sixty hazardous beer drinkers were recruited into the study, however one participant was lost to Day 8 testing such that the final N was fifty nine (Control N = 19, REACT+PE N = 20, REACT no PE N = 20). All participants reported drinking beer on at least four days of every week. Hazardous drinking was defined as scoring over 10 on the Alcohol Use Disorders Identification Test (AUDIT), but not meeting alcohol dependence criteria based on the Structured Clinical Interview for DSM-IV (SCID)([31](#_ENREF_31)). Mean age of participants was 22.6±6.07 years. Participants were reimbursed at the rate of £7.50 per hour. Participants were allocated to groups one of three groups: Control (receiving no beer memory reactivation prior to counterconditioning), Reactivation with prediction error (REACT + PE, who retrieved cue-beer memories with an explicitly guided prediction error prior to counterconditioning) and reactivation with no prediction error (REACT no PE; who retrieved cue- beer memories and consumed beer with no prediction error). Participant numbers were randomly assigned to different groups such that there were equal numbers in each group and participants allocated a participant number upon enrolment into the study.

Ethical approval for the study was provided by University College London Graduate School Ethics Committee.

**Stimuli:**

*CSs*

Four prototypical beer images were selected to act as MMM reactivation cues in the rating task and subsequently as CS+s in the counterconditioning task. These were obtained from internet media sources and depicted beer taps on a bar, a poured pint of beer, an ice bucket filled with beer bottles and a can of beer being poured into a pint glass, in order to represent the major modes of beer consumption. Two novel beer images, used on Day 8 in the liking (picture rating) and attentional bias task depicted pints of beer on a table and a pint of beer being consumed, two novel wine images depicted a glass of wine next to a bottle and a glass of wine being consumed. Two CS-s were used in the counterconditioning. These depicted a cup of coffee and a can of cola with a glass. Soft drink images were used as CS-s to assess generic decrease in liking of consumable stimuli due to anti-consummatory effects of exposure to the disgust UCSs. In the control group, the pictures rated during the retrieval stage depicted oranges, an orange being squeezed, a glass of orange juice and a woman consuming orange juice. The beer and orange juice cues were equated as much as possible to minimize any effects that were not specific to the reactivation manipulation. All stimuli were presented via 1024x768 pixel flat screen 21 inch monitor.

Multiple, prototypical beer CSs were used to maximize activation of MMM networks and generalization of the association between beer-related stimuli and disgusting outcomes. The use of single discrete stimuli in reconsolidation paradigms can lead to effects that are highly specific to that stimulus, rather than generalizing to novel stimuli within the class of the reactivated stimulus.

*UCSs*

Three highly arousing and negatively valenced UCS pictures were selected from the IAPS database based on content likely to elicit disgust ([32](#_ENREF_32)). These depicted a toilet covered in fecal matter (image 9301), a man vomiting (image 9325) and a badly wounded human hand (image 9405). A fourth picture sourced from the internet depicted a septic wound on a human foot that was infested with maggots. Disgusting drinks were made by mixing 80μL 2.5% Denatonium Benzoate (Bitrex solution) in 120ml water. This solution was divided into 8 15ml drinks. Bitrex was kindly provided free of charge by Macfarlan Smith Ltd (City, UK).

*Visual probe:*

All CSs used in the counterconditioning task on Day 1 were paired with composition and complexity-matched images (non-target images) that did not depict alcohol. All pictures in both the counterconditioning and visual probe task were 300 x 300 pixels.

*Subjective assessments:*

Hazardous alcohol use was confirmed with the Alcohol Use Disorders Identification Test (AUDIT)([33](#_ENREF_33)), with scores > 10 being acceptable for the study. Level of alcohol dependence was assessed with via DSM IV SCID criteria, with < 4 items coded at threshold level being acceptable. Drinking prior to Day 1 and after the day 1 intervention was assessed using an electronic version of the Timeline Follow Back for Alcohol (TLFB) ([26](#_ENREF_26)). Momentary craving for alcohol was assessed with the Alcohol Craving Questionnaire (ACQ-NOW)([30](#_ENREF_30)). Drinking concern and readiness to change were measured with the Stages Of Change Readiness And Treatment Eagerness Scale (SOCRATES) ([27](#_ENREF_27)).Propensity and sensitivity to disgust were measured via the Disgust Propensity and Sensitivity Scale Revised (DPSS-R) ([34](#_ENREF_34)) and expectancies of drinking-related outcomes were assessed with the Negative Alcohol Expectancy Questionnaire (NAEQ) ([35](#_ENREF_35))

**Procedure:**

Twenty four hours prior to attending the study center, participants completed and returned the SOCRATES and TLFB measures. TLFB was assessed for the week prior to the study and a daily average beer consumption was computed. These measures were completed prior to Day 1 to minimize the amount of alcohol memory retrieval immediately prior to the manipulation on Day 1.

*Day 1:*

Participants were not informed of the exact nature of the study, but rather were told that the experimenters were interested in taste perception and learning processes in heavy drinkers. As part of this, participants were told they would be required to consume different drinks samples, some of which might be very bitter. Participants were randomly allocated to one of three groups that differed only in the nature of the retrieval phase on Day 1. After providing written informed consent, participants completed the DPSS-R and immediately began the relevant retrieval phase of the retrieval/counterconditioning procedure.

*Memory retrieval:*

In the control condition, a 150ml glass of chilled orange juice was placed in front of the participants as the computer task began. In both the REACT+PE and REACT No P.E conditions, an identical glass of 150ml chilled non-alcoholic beer was placed in front of the participants. Participants were not informed of the non-alcoholic nature of the beer. On screen-instructions informed participants that the experiment would look at how viewing images affected their perception of the taste of drinks and that they would rate a series of images, then consume the drink in front of them. They were told that they were to consume the whole of the drink according to on-screen prompts, which were a sequence of screens displaying ‘PICK UP DRINK’ ‘PREPARE TO DRINK’ and ‘DRINK NOW’, each screen displayed for 2000ms. An example of these screens were given and participants were told only to drink when ‘DRINK NOW’ appeared on screen. Participants’ understanding of the instructions was confirmed before they began rating the images. The control group then rated the four orange juice images and two CS- images (coffee and cola) the REACT+PE and REACT no PE groups rated the four beer CSs and two CS-. Following the final rating, the drinking prompts began. All ratings were made on the scale 1(extremely unpleasant) to 10(extremely pleasant) via labelled keys on the keyboard. In the Control and REACT no P.E. groups, these screens proceeded as per the instructions and they consumed the beer or orange juice, as expected. In the BEER no PE group, this recapitulated a standard drinking episode, and therefore no new information was available to destabilize learning. In the REACT + PE group, the first two prompt screens were as expected, but the final screen unexpectedly displayed the words ‘STOP! DO NOT DRINK’, followed by ‘Put the drink down and alert the experimenter’. In all groups, the glass was then removed and the distractor tasks began.

*Distractor Tasks:*

Participants completed verbal and category fluency tests, digit span forwards and backwards ([36](#_ENREF_36)), Trail making version A (numeric) and B (alphanumeric)([37](#_ENREF_37)) and digit cancellation tasks. These distractor tasks were chosen due to their high attentional and working memory demands. As offset of reactivated stimuli is critical for the switch between memory reconsolidation and extinction, high working memory tasks prevented maintenance of rated stimuli in working memory during the 10 minute period between retrieval and counterconditioning. Performance in the distractor tasks was not of primary interest to the current study and is therefore not reported here.

*Counterconditioning:*

Counterconditioning began immediately after completion of the distractor tasks. On-screen instructions told participants that they would now continue rating pictures and consuming samples of drinks, some of which may be extremely bitter, but were completely harmless. As before, participants were instructed to consume the entire drink placed in front of them whenever the words ‘DRINK NOW’ appeared on the screen. Participants were told that an image would appear on one side of the screen and that an outcome image would appear on the other. This would either be a picture or the words ‘DRINK NOW’. Participants were required to make two ratings per trial; the first when the initial image appeared to rate its pleasantness and the second after offset of the outcome (after viewing the UCS image or consuming the drink) to rate the pleasantness of the outcome. Each CS was presented four times during the counterconditioning. Two of the beer CS+s were paired with the disgusting picture outcomes (each CS was paired once with each of the UCS images) and two of the beer CSs were followed by 15ml 0.067% aqueous Bitrex solution on a 100% reinforcement schedule.

A single, pseudo randomized trial order was used for all participants. Trials were randomized with the stipulation that the same UCSs could not occur in consecutive trials and that no more than two trials of the same CS could occur consecutively. Participants in the Control condition rated the four beer CSs at the beginning of the counterconditioning session. The purpose of this was firstly to provide a baseline measure of liking for these stimuli and secondly to ensure that identical numbers of CS presentations were given in each group so that effects could not be attributed to differential amounts of CS exposure. The eight Bitrex-containing drinks were stored in an opaque box, so that participants were unaware how many more drinks they were required to consume. New drinks were placed in front of participants immediately after consumption of the previous drink. At the end of the task, participants received two squares of milk chocolate to get rid of the taste of Bitrex.

*Day 8:*

Participants first completed the liking ratings of all the stimuli from Day 1. The novel beer and wine stimuli were also rated. They then completed the dot probe task to assess attentional bias towards alcohol-related stimuli.

*Visual* *probe*

Alcohol and matched non-target image pairs were presented 8 times each in a random order, balanced for laterality of target image (left or right), laterality of probe location (ipsilateral to target image, contralateral to target image) and probe orientation (pointing up or down). Eye movements during the task were tracked with an Eyelink 1000 desktop mounted eye tracker (SR Research, Ontario, Canada) with a sampling rate of 1KHz. Participants heads were stabilized in head mount set 60cm away from the computer screen throughout. Trials began with a drift correction, where central fixation was verified by the experimenter. Image pairs then appeared for 2000ms, after which the images disappeared and a triangular probe appeared in the location where one of the images had been. The triangle either pointed upwards or downwards and participants had to respond as to the orientation of the probe as quickly and accurately as possible.

These behavioral measures were simply to ensure continued engagement in the task as in practice they were superseded by eye-tracking which directly measures attentional allocation to stimuli. Attentional bias scores for each image pair were calculated as total time spent fixating on target image – total time fixating on matched control image, so positive scores indicate a bias towards target images and negative scores a bias away from them. Fixations occurring <100ms after image pair onset were excluded from dwell time calculation, as they represent pre-emptive looking to stimulus locations. After completing the visual probe, participants completed the ACQ-NOW, SOCRATES, DPSS-R and TLFB for the preceding week. This concluded the testing procedure.
